# Comparative Analysis of Cellular and Growth Factor Composition in Bone Marrow Aspirate Concentrate and Platelet-Rich Plasma

**DOI:** 10.1155/2018/1549826

**Published:** 2018-02-25

**Authors:** Hisashi Sugaya, Tomokazu Yoshioka, Toshiki Kato, Yu Taniguchi, Hiroshi Kumagai, Kojiro Hyodo, Osamu Ohneda, Masashi Yamazaki, Hajime Mishima

**Affiliations:** ^1^Division of Regenerative Medicine for Musculoskeletal System, Department of Orthopedics Surgery, Faculty of Medicine, University of Tsukuba, 1-1-1 Tennodai, Tsukuba, Ibaraki 305-8575, Japan; ^2^Laboratory of Regenerative Medicine and Stem Cell Biology, Graduate School of Comprehensive Human Sciences, University of Tsukuba, 1-1-1 Tennodai, Tsukuba, Ibaraki 305-8575, Japan; ^3^Department of Orthopedic Surgery, Faculty of Medicine, University of Tsukuba, 1-1-1 Tennodai, Tsukuba, Ibaraki 305-8575, Japan

## Abstract

The purpose of this study was to quantify the stem cell and growth factor (GF) contents in the bone marrow aspirate concentrate (BMAC) and platelet-rich plasma (PRP) prepared from whole blood using a protocol established in our laboratory. We examined 10 patients with osteonecrosis of the femoral head who were treated by autologous BMAC transplantation at our hospital between January 2015 and June 2015. We quantified CD34+ and CD31−CD45−CD90+CD105+ cells in BMAC and PRP by flow cytometry. Additionally, we measured various GFs, that is, basic fibroblast growth factor (b-FGF), platelet-derived growth factor-BB (PDGF-BB), vascular endothelial growth factor (VEGF), transforming growth factor-*β*1 (TGF-*β*1), and bone morphogenetic protein-2 (BMP-2) in BMAC and PRP using enzyme-linked immunosorbent assays and statistical analyses. CD34+ and CD31−45−90+105+ cells accounted for approximately 1.9% and 0.03% of cells in BMAC and no cells in PRP. The concentration of b-FGF was higher in BMAC than in PRP (*P* < 0.001), whereas no significant differences in the levels of PDGF-BB, VEGF, TGF-*β*1, and BMP-2 were observed between the two types of sample. BMAC had an average of 1.9% CD34+ and 0.03% CD31−45−90+105+ cells and higher levels of b-FGF than those of PRP.

## 1. Introduction

Bone marrow aspirate (BMA) is a mixture of aspirated BM cells, small tissue fragments, and venous sinus blood (peripheral blood). Its bone-stimulating effects were first described in experiments using rabbits [1]; it was later discovered that fibroblast-like cells with adhesive, colony-forming, and osteogenic potential are present in BM [2]. It is now known that mesenchymal stem cells in the BM have the potential to differentiate into various mesenchymal tissues [3]. BMAs have been used in orthopedic surgery for nonunion [4], the treatment of osteonecrosis (especially femoral head necrosis) and osseous defects, and bone lengthening [5–8].

We previously developed a treatment for osteonecrosis and nonunion using 30–40 ml of BMA concentrate (BMAC) produced without special instruments from 300–400 ml of BMAs harvested from the ilium and centrifuged [9–14]. To ensure that the buffy coat layer of the BMA was extracted without leakage, the areas immediately above and below this layer were also manually extracted. Thus, the BMAC also contained the platelet-rich plasma (PRP) formed just above the buffy coat layer. This fraction, which is a plasma fraction containing an abundance of platelets, has clinical applications in the field of orthopedic surgery as a regenerative treatment, primarily for sports injuries [15, 16]. Our earlier studies demonstrated the therapeutic value of PRP obtained from whole peripheral blood (WB) [16, 17]. Transplanting BMAC and PRP together also has positive results [18, 19]. However, BMAC and PRP preparation methods vary from one experiment to the next; as such, there are no conclusive results regarding differences between cellular components and humoral factors contained in BMAC or PRP.

To address this issue, we analyzed the stem cell and growth factor contents of the BMAC produced using our protocol. Specifically, we quantified hematopoietic progenitor/stem cell and mesenchymal stromal/stem cell populations as well as growth factor concentrations. We also evaluated differences in the compositions of BMAC and PRP prepared from WB.

## 2. Materials and Methods

All procedures were approved by the Institutional Ethics Review Committee of the University of Tsukuba. Informed consent was obtained preoperatively from all study participants.

### 2.1. Patients

Ten patients with osteonecrosis of the femoral head (ONFH) who were treated by concentrated autologous BMA transplantation at our hospital between January 2015 and June 2015 were included. The mean age of the eight male patients and two female patients was 40 years (range: 28–59 years). In this cohort, ONFH developed in eight patients following long-term corticosteroid use and in two patients who abused alcohol.

### 2.2. BM Aspiration and Concentration

BM was aspirated and concentrated according to a previously published method [9–14]. Briefly, patients were given general anesthesia and BM was aspirated from both anterior iliac crests using a BM harvest needle (Medical Device Technologies, Gainesville, FL, USA) with 20 ml syringes prefilled with 1.5 ml of the anticoagulant citrate dextrose. Aspirates were collected using a BM collection kit (Baxter, Deerfield, IL, USA) and then transferred to a quadruple blood bag (Terumo, Tokyo, Japan) for concentration by a two-step centrifugation method (KUBOTA 9800; Kubota, Tokyo, Japan). The blood bag contents were first centrifuged at 1200 ×g for 10 min at room temperature, and erythrocytes were removed from the original bag. The remaining contents were centrifuged at 3870 ×g for 7 min at room temperature, after which the plasma and anticoagulants were removed followed by extraction of the BMAC containing the buffy coat. This technique reduced the 400 ml volume of BMA to 30–40 ml of BMAC, of which 4 ml was used for experiments.

### 2.3. Preparation of PRP Derived from WB

At the time of BM aspiration and concentration, 18 ml of WB was obtained from the same patient. PRP was prepared from WB as previously described [20]. A 21-gauge butterfly needle was used to draw 18 ml of WB from the cubital vein of the subjects, while avoiding hemolysis. The blood was collected in two 9 ml Spitz tubes containing 3.8% citric acid and was centrifuged at 580 ×g for 8 min at room temperature (PRGF-Endoret IV System; BTI Biotechnology Institute, Vitoria, Spain). The contents of each tube were separated into the erythrocyte, buffy coat, and plasma layers. In the separated plasma layer, a safety area was delineated in the upper area of the buffy coat, while avoiding aspirating the buffy coat; the upper and lower halves of the plasma layer were defined as platelet-poor plasma and PRP, respectively. About 2 ml of PRP per Spitz tube was collected by aspiration using a dedicated aspirator (PRGF-Endoret IV; BTI Biotechnology Institute) for a total of about 4 ml of PRP from the two tubes.

### 2.4. Hematological Analysis

Nucleated white blood cells and platelets in BMA, BMAC, WB, and PRP were counted using an automated system (Sysmex KX-21N; Sysmex Corp., Kobe, Japan).

### 2.5. Fibroblastic Colony-Forming Unit (CFU-F) Assay and Cell Characterization

To determine the presence and proportion of progenitor cells in BMA and BMAC, samples from each patient were evaluated with the CFU-F assay, as previously described [10, 12, 14]. The samples (100 *μ*l) were washed twice with phosphate-buffered saline and then resuspended in 3 ml of growth medium composed of Dulbecco's Modified Eagle's Medium (Sigma-Aldrich, St. Louis, MO, USA) supplemented with 10% fetal bovine serum (Gibco, Grand Island, NY, USA) and antibiotic–antimycotic solution (Gibco). The cells were seeded in 60 cm^2^ dishes and cultured at 37°C in a humidified atmosphere of 5% CO_2_. The medium was replaced 2 days later and nonadherent cells were removed; thereafter, the medium was replaced twice a week. After 2 weeks, the medium was removed and cells were stained with 0.5% Crystal Violet (Sigma-Aldrich) in methanol for 5 min and washed twice with distilled water before the number of CFU-Fs was counted. Colonies of <2 mm in diameter and those that were only faintly stained were excluded. The number of progenitor cells is expressed as the number of CFU-Fs per 10^6^ nucleated cells.

In order to analyze the cellular composition of BMAC, the number of CD34+ cells, which are precursors of hematopoietic cells, and CD31−45−90+105+ cells were determined. BM cells in BMAC were incubated in red blood cell lysis buffer (VersaLyse; Beckman Coulter, Brea, CA, USA) at 37°C for 5 min, followed by incubation for 30 min at 4°C with fluorescein isothiocyanate-conjugated anticluster of differentiation (CD)31 (1 : 40), phycoerythrin (PE)/Cy5-conjugated anti-CD90 (1 : 200), energy-coupled dye (PE-Texas Red)-conjugated anti-CD45 (1 : 40), and allophycocyanin- (APC-) conjugated anti-CD105 (1 : 200) antibodies (all from BD Pharmingen, San Jose, CA, USA), and APC-conjugated anti-CD34 antibody (1 : 200; Biolegend, San Diego, CA, USA). Matching isotype control antibodies were used as a control. After incubation, labeled cells were sorted on a Gallios flow cytometer (Beckman Coulter), and data were analyzed using Kaluza software (Beckman Coulter).

### 2.6. Growth Factor (GF) Quantification

The BMAC and PRP were incubated at 37°C for 1 h after adding 5% calcium chloride (BTI Biotechnology Institute) and centrifuged at 1000 ×g for 20 min at 4°C. The supernatant was removed and stored at −80°C. The cryopreserved supernatant was thawed at room temperature before use. The concentrations of basic fibroblast growth factor (b-FGF), platelet-derived growth factor- (PDGF-) BB, transforming growth factor- (TGF-) *β*1, vascular endothelial growth factor (VEGF), and bone morphogenetic protein- (BMP-) 2 were measured using enzyme-linked immunosorbent assay kits specific for each GF (R&D Systems, Minneapolis, MN, USA) according to the manufacturer's recommendations. All standards and samples were analyzed in duplicate.

### 2.7. Statistical Analysis

The numbers of nucleated cells and platelets in BMAC and PRP before and after the concentration procedure were analyzed using Wilcoxon's rank sum tests. The GF quantification results for BMAC and PRP were analyzed using Mann–Whitney *U* tests. *P* < 0.05 was considered statistically significant. Analyses were performed using IBM SPSS Statistics v.24.0 (IBM Corp., Armonk, NY, USA).

## 3. Results

### 3.1. Characterization of Cells in BMAC and PRP

**Figure 1:**
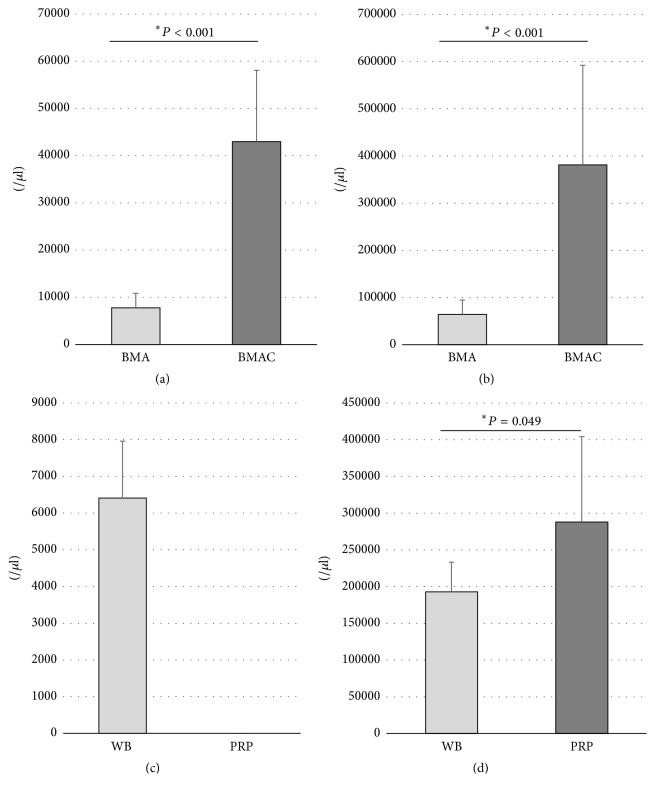
Numbers of nucleated cells and platelets in BMAC and PRP before and after concentration. (a) BMAC had higher levels of WBC than BMA (*P* < 0.001). (b) BMAC had higher levels of PLT than BMA (*P* < 0.001). (c) No nucleated cells were detected in PRP samples. (d) PRP had higher levels of PLT than WB (*P* = 0.049). (^*∗*^*P* < 0.05).

The volume of BMA obtained was 402 ± 35 ml; after concentration, the volume was 35.4 ± 1.6 ml. The average ratio of the volumes after and before concentration was 11.4. The findings of the hematological analysis are presented in Figure 1. On average, the number of nucleated cells was 5.53-fold higher in BMAC (43.0 ± 15.1  × 10^3^/*μ*l) than in WB (7.78 ± 3.06  × 10^3^/*μ*l), and BMAC had higher levels of WBC than BMA (*P* < 0.001). The average platelet count was also 5.93-fold higher in BMAC (38.1 ± 21.1  × 10^4^/*μ*l) than in BMA (6.42 ± 3.06  × 10^4^/*μ*l) and BMAC had higher levels of PLT than BMA (*P* < 0.001). No nucleated cells were detected in PRP samples. The average platelet count was 1.49-fold higher in PRP (28.8 ± 11.6  × 10^4^/*μ*l) than in WB (19.3 ± 4.04  × 10^4^/*μ*l) and PRP had higher levels of PLT than WB (*P* = 0.049). Based on platelet counts and the presence of white blood cells and neutrophils, the PRP preparation was classified as P2-x-B*β* according to the platelet-activation white blood cell classification system [21].

**Figure 2:**
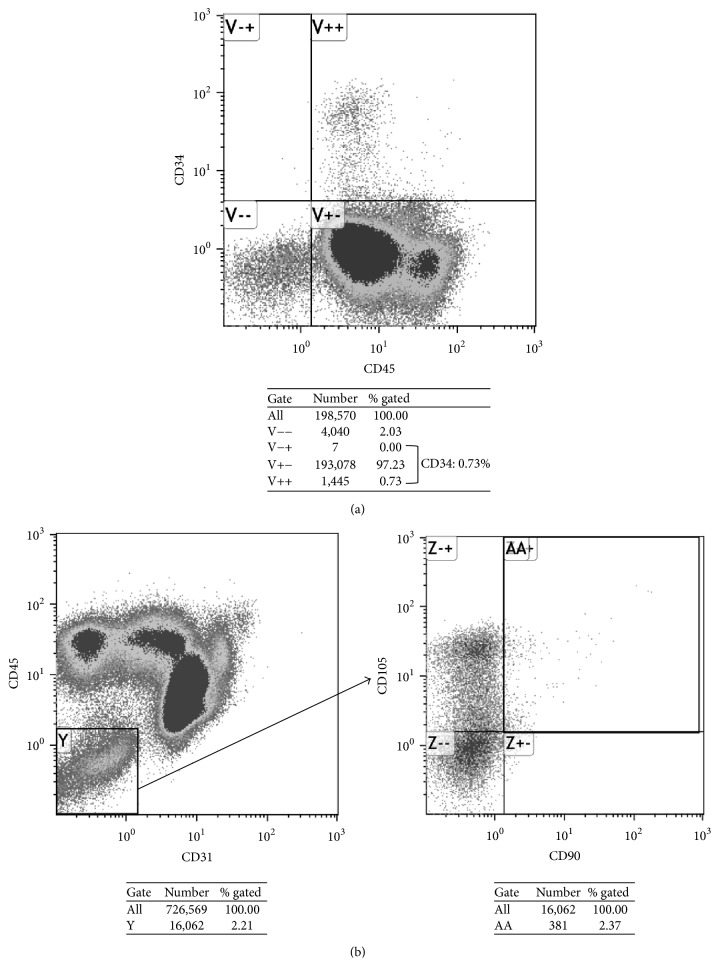
Flow cytometry results for CD34+ cells and CD31−45−90+105+ cells. Results for a 37-year-old woman with corticosteroid-induced ONFH. (a) The percentage of CD34+ cells per total BM cells was 0.73% (1,452/198,570). (b) The percentage of CD31−45−90+105+ cells per total BM cells was 0.052% (381/726,569).

The mean CFU-F count in BMAC (196 ± 161/ml) was higher than that in BMA (31.6 ± 37.0/ml). The mean concentration rate of CFU-F was 6.0 ± 4.4, and the number of CFU-F per 10^6^ nucleated cells in BMAC was 4.62 ± 4.07. The mean percentage of CD34+ cells per total BM cells was 1.87% (range: 0.60%–4.25%), and the mean percentage of CD31−45−90+105+ cells per total BM cells was 0.030% (range: 0.001%–0.101%) (Figure 2).

### 3.2. GF Levels in BMAC and PRP

**Figure 3:**
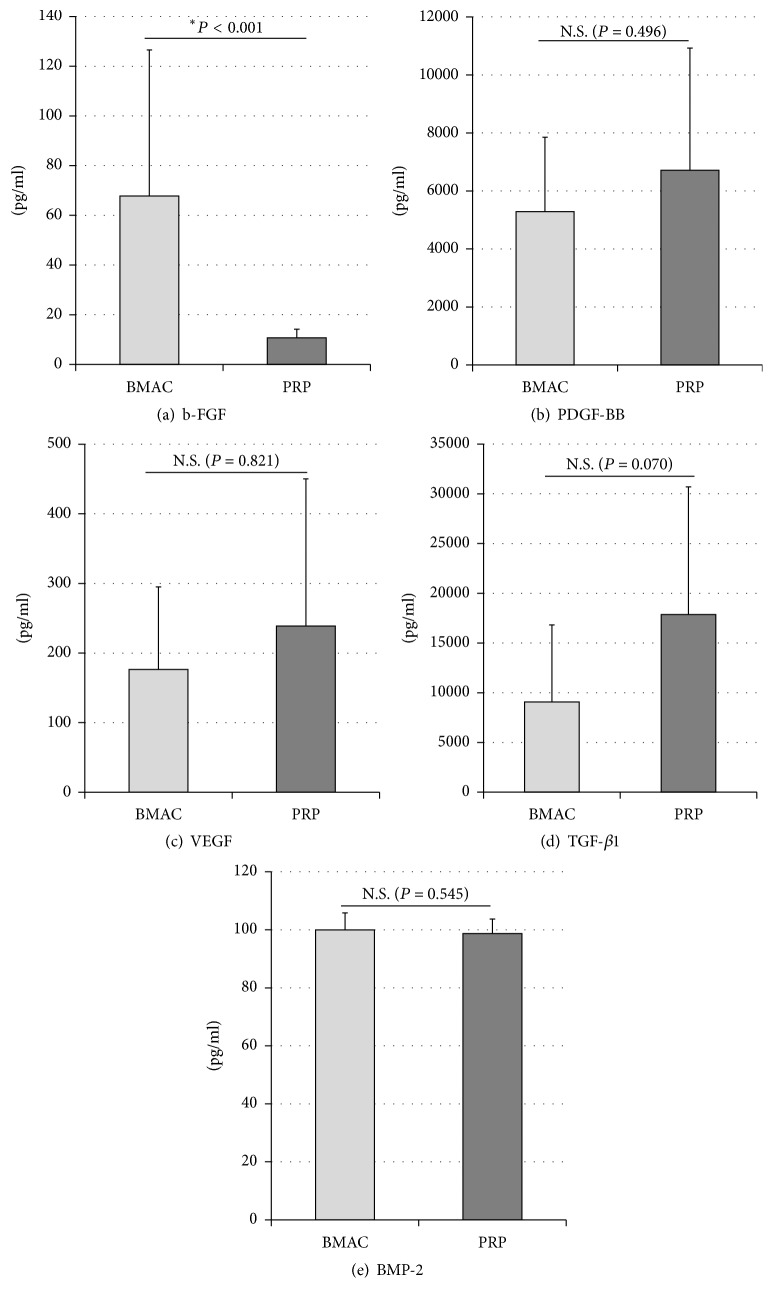
GF levels in BMAC and PRP. (a) BMAC had higher levels of b-FGF than PRP (*P* < 0.001). (b–e) There were no differences between PRP and BMAC in terms of PDGF-BB, VEGF, TGF-*β*1, and BMP-2 levels. (^*∗*^*P* < 0.05).

**Figure 4:**
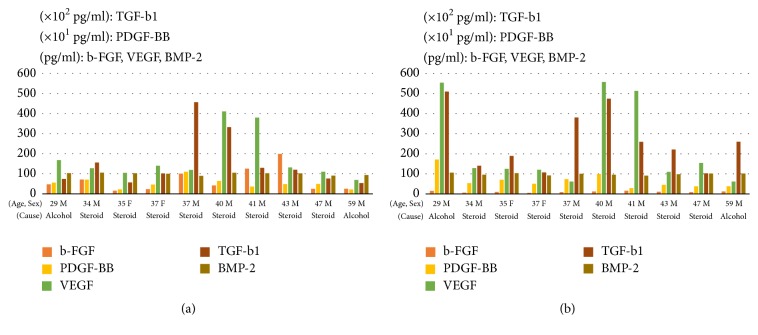
GF levels for each patient. (a) BMAC; (b) PRP.

GF levels in BMAC were as follows: b-FGF, 6.78 ± 5.87  × 10^1^ pg/ml; PDGF-BB, 5.28 ± 2.57  × 10^3^ pg/ml; VEGF, 1.76 ± 1.18  × 10^2^ pg/ml; TGF-*β*1, 1.56 ± 1.33  × 10^4^ pg/ml; and BMP-2, 9.99 ± 0.59  × 10^1^ pg/ml. The levels in PRP were as follows: b-FGF, 1.07 ± 0.35  × 10^1^ pg/ml; PDGF-BB, 6.71 ± 4.21  × 10^3^ pg/ml; VEGF, 2.39 ± 2.11  × 10^2^ pg/ml; TGF-*β*1, 2.65 ± 1.46  × 10^4^ pg/ml; and BMP-2, 9.87 ± 0.50  × 10^1^ pg/ml. The concentration of b-FGF was higher in BMAC than in PRP (*P* < 0.001), whereas no significant differences in the levels of the other GFs were observed between the two types of sample (Figure 3). GF levels for each patient are presented in Figure 4.

## 4. Discussion

CD34+ and CD31−45−90+105+ cells accounted for approximately 1.9% and 0.03% of cells in BMAC, respectively. BMAC contained a higher concentration of b-FGF than PRP, although the two types of sample had similar concentrations of the other GFs. It was previously demonstrated that CD34+ positive cells make up about 1% of the total number of mononuclear cells [22]; other investigators have reported that the proportion of nucleated cells in BMAC that were CD34+ was 1.0% ± 0.2% [23], which is consistent with our observations.

CFU-Fs constitute 0.01%–0.001% of all BM mononuclear cells [24], corresponding to a mesenchymal stem cell (MSC) ratio of 1/100,000 hematopoietic cells [25]. Although the average number of CD31−45−90+105+ cells was relatively high (0.03%), the average number of CFU-Fs was small (4/10^6^ mononuclear cells). Other investigators have obtained results similar to those of the present study using the same BMAC preparation method, with an average number of CFU-Fs of 2.55/10^6^ mononuclear cells [14]. On the other hand, a CD45−CD271+ cell fraction of 0.016% (from 0.009%–0.032%) was observed in BMAs, with 1520 cells per 1 ml of BM (96–20,992) and a significantly lower average CFU-F count, that is, 60 (3–900) per 1 ml of BM [26] or comparable results [27, 28]. In the present study, we also obtained a high cell count based on an analysis of cell surface antigen expression as compared to CFU-F.

To measure GF levels in BMAC and PRP, 5% calcium chloride was added to both samples, that is, BMAC and PRP, for platelet activation. Sánchez et al. reported that calcium chloride leads to platelet activation and the hydrolysis of prothrombin into thrombin, which simultaneously causes the release of myriad growth factors and the polymerization of fibrin [20].

A comparison of GF levels in BMAC and WB PRP revealed no significant differences in PDGF, TGF-*β*1, interleukin- (IL-) 8 or interleukin-6, tumor necrosis factor-*α*, interferon-*γ*, or FGF-1 levels; however, VEGF and IL-1 receptor antagonist levels were higher in BMAC than in PRP [29]. We found similar results for PDGF and TGF-*β*1 but observed no significant differences in VEGF expression, whereas b-FGF was elevated in BMAC as compared to PRP.

VEGF modulates angiogenesis, migration, and mitosis of endothelial cells as well as the creation of the blood vessel lumen [30, 31]. The PRP VEGF concentration of 2.39 ± 2.11  × 10^2^ pg/ml obtained here is comparable to the value of 2.76 ± 2.73  × 10^2^ pg/ml found in a previous study [29]. However, others have reported VEGF concentrations in PRP of 76–854 pg/ml [30] and 10 ± 12 pg/ml [29]. Moreover, we found a BMAC VEGF concentration of 1.76 ± 1.18  × 10^2^ pg/ml, which is higher than that reported by Cassano et al. [29], but comparable to the results of Alsousou et al. [30] (Table 1). Our data suggest that PRP and BMAC contain equivalent amounts of VEGF. However, given that this is contradicted by other reports, a more detailed analysis of a larger number of cases is warranted.

FGF belongs to a family of heparin-binding polypeptide GFs that includes b-FGF (FGF-2), which activates signaling pathways involved in the development and maintenance of cartilage [32]. b-FGF is known to induce cell proliferation and chondrogenic differentiation in human BM MSCs [33]. However, there are no studies of b-FGF concentrations in BMAC; in one study, FGF-1 was not detected in BMAC or PRP [29], but b-FGF was not evaluated. We found that b-FGF was present at higher concentrations in BMAC than in PRP.

b-FGF is involved in the maintenance of the MSC cytoplasmic volume and spindle morphology [34–36]. MSCs cultured with FGF maintained the spindle shape typical of fibroblasts, whereas, in the absence of FGF, cells developed a large cytoplasmic volume [34]. Identical results were obtained for MSCs derived from human BMAs [35–37]. In this study, we found MSCs in BMAC, but not in PRP, and observed higher concentrations of b-FGF in BMAC; however, it remains to be determined whether b-FGF plays a role in maintaining MSC cell morphology.

Using our method, leukocytes were not detected in PRP, and the platelet counts in PRP were lower than those for other methods. Some studies have reported platelet or leukocyte counts in PRP and GF levels. In PRP prepared using five different systems, Magalon et al. found positive correlations between platelet counts in PRP and levels of VEGF, PDGF-AB, and TGF-*β*1 and between leukocyte counts in PRP and levels of VEGF [38]. Zimmermann et al. reported remarkable interindividual variation in GF levels and no correlation between GF levels and platelet concentration [39]. Castillo et al. reported a positive correlation between GF levels and leukocyte counts in PRP [40]. Our method might have a disadvantage with respect to GF levels in PRP owing to the relatively low enrichment for platelets.

These findings are applicable to the procedures and instruments that we developed to obtain BMAC and PRP; similar tests, including comparative analyses, should be performed when other methods are used for sample preparation. One limitation of this study was the small sample size. For patients with ONFH, blood sampling to obtain PRP was not directly related to their treatment and accordingly the number of samples was limited. Furthermore, since BMAC and PRP preparation methods can vary, caution must be exercised when comparing the results of different studies.

## 5. Conclusion

We produced BMAC that contained an average of 1.9% CD34+ and 0.03% CD31−45−90+105+ cells. BMAC had higher levels of b-FGF than PRP, although the two samples had comparable levels of PDGF-BB, VEGF, TGF-*β*1, and BMP-2. These findings suggest that our protocol can be used to produce BMAC for therapeutic applications, such as the treatment of osteonecrosis and nonunion.

## Figures and Tables

**Table 1:** Comparison of VEGF levels in BMAC and PRP.

	VEGF level (pg/ml)	
	BMAC	PRP
Our study	239 ± 211	176 ± 118
Cassano et al. [29]	276 ± 273	10 ± 12
Alsousou et al. [30]	-	76 to 854
